# The clinical evaluation value of the NRF2-mediated antioxidant stress mechanism in elderly patients with type 2 diabetes mellitus complicated by osteoporosis

**DOI:** 10.5937/jomb0-58290

**Published:** 2026-02-27

**Authors:** Reyila Fulati, Guzainu Ailiyasi, Renagu Baihetiya, Jiwei Yu, Xiufang Wang, Zhenhua Li, Gulizhaer Tulake

**Affiliations:** 1 The First People's Hospital of Kashi, Department of Geriatric, Kashi City, Xinjiang Uygur Autonomous Region, China; 2 The First People's Hospital of Kashi, Department of Endocrinology, Kashi City, Xinjiang Uygur Autonomous Region, China; 3 The First People's Hospital of Kashi, Department of Rheumatology and Immunology, Kashi City, Xinjiang Uygur Autonomous Region, China

**Keywords:** NRF2, oxidative stress, type 2 diabetes mellitus, osteoporosis, NRF2, oksidativni stres, dijabetes melitus tipa 2, osteoporoza

## Abstract

**Background:**

The role of nuclear factor erythroid 2-related factor 2 (NRF2) as a central transcription factor in the regulation of cellular antioxidant stress is of significant interest, particularly in its potential clinical relevance for elderly patients with type 2 diabetes mellitus (T2DM)-associated osteoporosis (T2DM-OP).

**Methods:**

A cohort of 196 patients with T2DM who received treatment were recruited and stratified into three groups according to T-scores derived from bone mineral density (BMD) assessments. Serum concentrations of 25-hydroxyvitamin D3, osteocalcin (OC), procollagen type I N-terminal propeptide (P1NP), b-isomerized C-terminal telopeptide of type I collagen (b-CTX), as well as nuclear factor erythroid 2-related factor 2 (NRF2), superoxide dismutase (SOD), and malondialdehyde (MDA) were quantified utilizing enzyme-linked immunosorbent assay techniques. Pearson correlation analysis was conducted to explore the relationships among NRF2, SOD, MDA, and BMD. Additionally, multivariate logistic regression analysis was employed to investigate the association between Asprosin levels and the risk of T2DM-OP.

**Results:**

There were no apparent differences in baseline characteristics (gender, age, BMI, duration of diabetes mellitus, and metabolic parameters) among the three groups (P &gt; 0.05). Analysis revealed that lumbar spine BMD decreased evidently with declining bone mass (normal bone mass group 1.13 g/cm2, osteopenia group 0.93 g/cm2, osteoporosis group 0.80 g/cm2, P &lt; 0.001). In the osteoporosis cohort, there was a significant elevation in the bone resorption marker b-CTX and the bone formation marker OC (P &lt; 0.001), indicating a pronounced imbalance in bone turnover. Among oxidative stress factors, SOD activity and NRF2 levels progressively decreased with bone loss, while MDA levels increased (all P &lt; 0.001), indicating declined antioxidant capacity and aggravated oxidative damage. Correlation analysis showed that NRF2 and SOD were positively correlated with BMD, whereas MDA was negatively correlated with BMD. Multivariate logistic regression further confirmed that low NRF2, low SOD, and high MDA were independent risk factors for T2DM-OP (P &lt; 0.05). Receiver operating characteristic curve analysis demonstrated that NRF2 exhibited high sensitivity (80.36%) and specificity (77.86%) in predicting T2DM-OP with an area under the curve of 0.856 (95% CI: 0.803-0.909, P &lt; 0.001), comparable to bone turnover indicators.

**Conclusions:**

The study reveals that the NRF2-mediated antioxidant response is crucial in the pathological progression of T2DM-OP highlighting its potential as a new therapeutic target for prevention and treatment.

## Abstract (sr)

**Uvod:**

Uloga faktora 2 povezanog sa nuklearnim faktorom eritroidom 2 (NRF2) kao centralnog faktora transkripcije u regulaciji ćelijskog antioksidativnog stresa je od značajnog interesa, posebno zbog njegovog potencijalnog kliničkog značaja za starije pacijente sa osteoporozom povezanom sa dijabetes melitusom tipa 2 (T2DM) (T2DM-OP).

**Metode:**

Kohorta od 196 pacijenata sa T2DM koji su prima li lečenj'e j'e regrutovana i stratifikovana u tri grupe prema T-skorima izvedenim iz procene mineralne gustine kostij'u (BMD). Koncentracij'e u serumu 25-hidroksivitamina D3, osteokalcina (OC), prokolagena tipa I N-terminalnog propeptida (P1NP), b-izomerizovanog C-terminalnog telopeptida kolagena tipa I (b-CTKS), kao i nuklearnog faktora povezanog sa eritroidom 2 faktora 2 (NRF2), dismuta i superoksida MDAS (NRF2), bile su dis-muta i malondioksid (NRF2) kvantifikovani korišćenj'em tehnika imunosorbentnog testa vezanog za enzime. Pir-sonova korelaciona analiza je sprovedena da bi se istražili odnosi između NRF2, SOD, MdA i BMD. Pored toga, korišćena je multivarijantna logistička regresiona analiza da bi se ispitala povezanost između nivoa asprosina i rizika od T2DM-OR.

**Rezultati:**

Nije bilo očiglednih razlika u osnovnim karakte-ristikama (pol, starost, BMI, trajanje dijabetes melitusa i metabolički parametri) među tri grupe (P &gt; 0,05). Analiza je otkrila da se BMD lumbalnog dela kičme evidentno smanjuje sa opadanjem koštane mase (grupa normalne koštane mase 1,13 g/cm2, grupa osteopenije 0,93 g/cm2, grupa osteoporoze 0,80 g/cm2, P &lt; 0,001). U kohorti osteoporoze, došlo je do značajnog povećanja markera resorpcije kosti b-CTKS i markera formiranja kostiju OC (P &lt; 0,001), što ukazuje na izraženu neravnotežu u koštanom prometu. Među faktorima oksidativnog stresa, aktivnost SOD i nivoi NRF2 progresivno su opadali sa gubitkom koštane mase, dok su se nivoi MDA povećali (svi P &lt; 0,001), što ukazuje na smanjeni antioksidativni kapacitet i pogoršano oksidativno oštećenje. Korelaciona analiza je pokazala da su NRF2 i SOD u pozitivnoj korelaciji sa BMD, dok je MDA u negativnoj korelaciji sa BMD. Multivarijantna logistička regresija je dalje potvrdila da su nizak NRF2, nizak SOD i visok MDA nezavisni faktori rizika za T2DM-OP (P &lt;0,05).

**Zaključak:**

Studija otkriva da je antioksidativni odgovor posredovan NRF2 ključan u patološkoj progresiji T2DM-OP naglašavajući njegov potencijal kao novi terapijski cilj za pre-venciju i lečenje.

## Introduction

With the accelerating global aging population, type 2 diabetes mellitus (T2DM) and osteoporosis (OP) have emerged as two highly prevalent chronic diseases with increasing comorbidity among the elderly [1][2]. Epidemiological data indicate that the incidence of OP in patients with T2DM is 2-3 times higher than in non-diabetic individuals [3]. A recent systematic review [4], reported that 37.8% of Chinese patients with T2DM suffer from OF, while patients with T2DM exhibit low bone turnover and bone formation rates [5]. The metabolic disturbances and chronic low-grade inflammation induced by prolonged hyperglycemia in patients with T2DM not only accelerate pancreatic β-cell dysfunction but also impair bone remodeling through complex molecular mechanisms. These pathological changes lead to reduced bone mass, deteriorated bone quality, and ultimately a significantly elevated risk of osteoporotic fractures [6]. Currently, no targeted therapy exists to effectively control the progression of T2DM-associated OP (T2DM-OP), highlighting the urgent need to identify novel therapeutic targets. Therefore, elucidating the pathological mechanisms underlying T2DM-OP and discovering biomarkers with dual potential for disease monitoring and therapeutic intervention have become clinical challenges requiring immediate resolution.

The unified mechanism hypothesis suggests that oxidative stress underlies the entire pathological process of diabetes and its complications, serving as a fundamental contributor to disease onset [7]. The pathogenesis of T2DM-OP is complex, where hyperglycemia may exacerbate bone cell dysfunction by amplifying systemic oxidative stress—a key etiological factor in T2DM complications [8][9]. Studies in estrogen-deficient mice demonstrate that oxidative stress elevates tumor necrosis factor- production, accelerating bone loss [10]. Nuclear factor-erythroid 2-related factor 2 (NRF2), a transcription factor involved in cellular antioxidant responses, contributes to maintaining redox homeostasis [11]. Under physiological conditions, NRF2 remains in an inactive state through its interaction with Kelch-like ECH-associated protein 1 (Keap1). Upon the onset of oxidative stress, NRF2 dissociates from Keap1 and translocates to the nucleus, where it induces the expression of antioxidant genes such as heme oxygenase-1 (HO-1) and superoxide dismutase (SOD). This activation plays a crucial role in mitigating reactive oxygen species (ROS) and attenuating cellular damage [12][13]. A recent study has found that the NRF2 signaling pathway not only participates in antioxidant defense but also influences bone homeostasis by regulating bone cell metabolism, inflammatory responses, and mitochondrial function. For instance, NRF2 can reduce high glucose-induced ferroptosis in bone cells by activating HO-1 and also promote bone formation by modulating the bone morphogenetic protein signaling pathway [14].

Building upon the aforementioned evidence, this study sought to systematically assess the expression profile of the NRF2 signaling pathway in elderly patients with T2DM-OP, and to elucidate its correlations with bone metabolism indicators and oxidative stress markers. Additionally, the research aimed to explore the potential efficacy of NRF2-targeted intervention strategies in enhancing bone health in this patient population. The findings are expected to yield new insights into the pathogenesis of T2DM-OP and establish a theoretical foundation for the development of targeted therapeutic strategies.

## Materials and methods

### Study subjects

A total of 196 elderly patients with T2DM who were hospitalized for the first time at The First People's Hospital of Kashi between January 1, 2023, and June 30, 2024, were prospectively enrolled in this study. Inclusion criteria: (1) Patients age ≥ 60 years; (2) Patients diagnosed with T2DM according to the 1999 the World Health Organization diagnostic criteria for diabetes mellitus [15]; (3) Patients with T2DM who had undergone BMD measurement using dual-energy X-ray absorptiometry; (4) Patients with normal cognitive function; (5) Patients who had not received hormone therapy within three months prior to enrollment in the study. Exclusion Criteria: (1) Patients with acute diabetes mellitus complications such as diabetic ketoacidosis, hyperosmolar hyperglycemic syndrome, or lactic acidosis; (2) Patients with abnormal liver or kidney function; (3) Patients with malignant tumors; (4) Patients with parathyroid disorders; (5) Patients who had taken medications affecting bone metabolism—such as sex hormones, glucocorticoids, vitamin D, or calcium supplements— within the past six months; (6) Patients with incomplete data. This study complies with medical ethics requirements and has been approved by the Medical Ethics Committee of The First People's Hospital of Kashi.

### BMD measurement and patient grouping

All subjects underwent lumbar spine (L1-L4) BMD and bone mineral content measurements using a dual-energy X-ray absorptiometry scanner (Norland Products, Inc., Connecticut, USA). T-scores were calculated accordingly. Subjects were categorized as follows: Normal bone mass group: T > -1.0; Osteopenia group: -2.5 < T ≤ -1.0; Osteoporosis group: T ≤ -2.5 [16]. According to the 1994 World Health Organization diagnostic standards, OP was defined as T < -2.5 with one or more fractures present.

### Blood sample collection and processing

Subjects underwent a fasting period exceeding 12 hours commencing the evening prior to the experimental procedure. On the subsequent morning, 5 mL of fasting peripheral venous blood was collected from each participant. The samples were allowed to equilibrate at room temperature for 30 minutes prior to centrifugation at 3,300 rpm for 10 minutes at 4°C. The resulting supernatant was then stored at -80°C for future analysis. Biochemical parameters, including fasting blood glucose (FBG), glycated hemoglobin (HbA1c), oral glucose tolerance test, and serum creatinine, were measured using an automated biochemical analyzer. Serum levels of nuclear factor erythroid 2-related factor 2 (NRF2), superoxide dismutase (SOD), malondialdehyde (MDA), 25-hydroxyvitamin D3 [25-(OH)D3], and bone metabolism markers-osteocalcin (OC), procollagen type I N- terminal propeptide (P1NP), and β-isomerized C-terminal telopeptide of type I collagen ((β-CTX)- were determined via enzyme-linked immunosorbent assay (ELISA). The ELISA kit for NRF2 was obtained from Abcam Limited (Cambridge, England), while kits for SOD, MDA, 25-(OH)D3, OC, P1NP and β-CTX were acquired from Nanjing Jiancheng Bioengineering Institute (Nanjing, China). All procedures were conducted in strict accordance with established protocols.

### Statistical analysis

Statistical analysis was performed using SPSS 24.0 software. The Shapiro-Wilk test was used to assess normal distribution of data. For normally distributed data, an independent samples t-test was used for comparisons between two groups; while one-way analysis of variance was employed for multiple group comparisons, with Welch's correction applied for data not showing homogeneity of variance, followed by LSD-t test for post-hoc analysis. Non-normally distributed data were expressed as M (P_25_, P_75_) and analyzed using Mann-Whitney U test for two-group comparisons and the Kruskal-Wallis H test for three-group comparisons. Categorical data were presented as counts (%) and compared using chi-square test. Pearson correlation analysis was conducted to examine relationships between NRF2-related oxidative stress indicators and BMD. Logistic regression analysis was performed to evaluate the risk of T2DM-OP associated with NRF2, SOD, and MDA. A P-value <0.05 was considered statistically significant.

## Results

### Comparison of baseline characteristics among the three groups

This study enrolled 196 participants (median age: 67.5 years; range: 64-72 years), including 104 male and 92 female individuals. Patients diagnosed with type 2 diabetes mellitus (T2DM) were categorized into three subgroups according to bone mineral density (BMD)-derived diagnostic criteria: normal BMD subgroup (n = 78), osteopenia subgroup (n = 62), and osteoporosis subgroup (n = 56). Comparative analysis of baseline demographic and metabolic profiles revealed no intergroup differences (P > 0.05) in gender distribution, age, BMI, diabetes duration, or metabolic parameters such as FBG, HbA1c, postprandial glucose, and lipid profiles (total cholesterol, triglycerides, HDL-C, and LDL-C) (Table 1).

**Table 1:** Comparison of baseline characteristics among the three groups.

Indicators	Normal bone mass group<br>(n = 78)	Osteopenia group<br>(n = 62)	Osteoporosis group<br>(n = 56)	*P*-value
Age	66.5 (64, 70)	68 (64, 72)	68.5 (65, 73.5)	0.127
Sex				0.480
Male	43 (55.13%)	29 (46.77%)	32 (57.14%)	
Female	35 (44.87%)	33 (53.23%)	24 (42.86%)	
BMI (kg/m^2^)	25.72 ± 2.18	25.09 ± 2.12	24.95 ± 2.08	0.080
Duration of T2DM (years)	9 (8, 11)	11 (8, 13)	10.5 (9, 14)	0.0652
FBG (mmol/L)	8.57 ± 1.04	8.60 ± 1.23	8.90 ± 1.35	0.208
HbA1c (%)	8.63 ± 1.09	8.80 ± 0.78	8.98 ± 0.82	0.101
2-hour postprandial blood<br>glucose (mmol/L)	15.23 ± 2.74	15.78 ± 2.63	15.94 ± 2.86	0.282
TC (mmol/L)	4.60 ± 0.99	4.95 ± 1.08	4.65 ± 1.09	0.122
TG (mmol/L)	1.81 ± 0.61	1.74 ± 0.54	1.78 ± 0.56	0.774
HDL (mmol/L)	1.05 ± 0.27	1.04 ± 0.29	1.08 ± 0.25	0.708
LDL (mmol/L)	2.40 ± 0.59	2.48 ± 0.54	2.39 ± 0.53	0.615

### Comparison of BMD and bone turnover marker levels among the three groups

**Figure 1:**
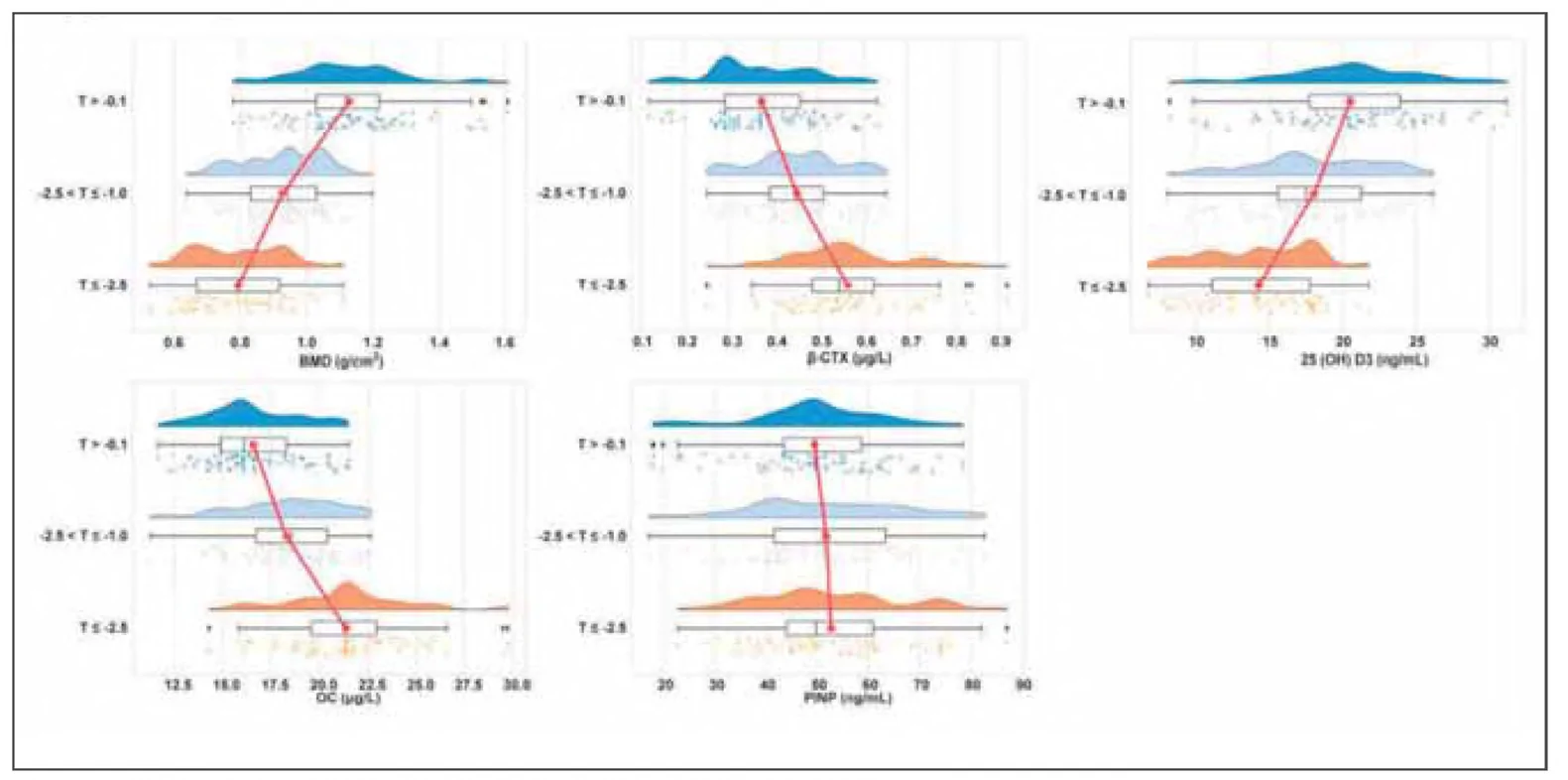
BMD and bone turnover marker levels among the three groups.

A comparative analysis was conducted to evaluate lumbar spine BMD and bone turnover marker levels across three distinct patient groups. The findings revealed a significant reduction in lumbar spine BMD correlating with progressive bone loss, with mean BMD values of 1.13 g/cm^2^ in the normal bone mass group, 0.93 g/cm^2^ in the osteopenia group, and 0.80 g/cm^2^ in the osteoporosis group (P < 0.001). This trend directly reflects the extent of bone mass reduction. Additionally, the osteoporosis group exhibited elevated levels of the bone resorption marker β-CTX and the bone formation marker OC, with mean values of 0.56 μg/L and 21.36 μg/L, respectively (both P < 0.001), indicating active yet imbalanced bone metabolism characteristic of high-turnover osteoporosis. Furthermore, vitamin D levels [25(OH)D3] significantly declined with increasing bone loss, with the lowest mean level observed in the osteoporosis group (14.28 ng/mL; P < 0.001), potentially contributing to the reduction in bone mass. Conversely, the bone formation marker PINP did not exhibit significant differences among the three groups (P = 0.391) (Table 2 and Figure 1).

**Table 2:** Comparison of BMD and bone turnover marker levels among the three groups.

Indicators	Normal bone mass<br>group (n = 78)	Osteopenia group<br>(n = 62)	Osteoporosis group <br>(n = 56)	*P*-value
Lumbar spine BMD (g/cm^2^)	1.13 ± 0.17	0.93 ± 0.13	0.80 ± 0.14	< 0.001
b-CTX (μg/L)	0.37 ± 0.11	0.45 ± 0.10	0.56 ± 0.13	< 0.001
25(OH)D_3_ (ng/mL)	20.54 ± 4.79	18.05 ± 4.35	14.28 ± 3.72	< 0.001
OC (μg/L)	16.59 ± 2.40	18.30 ± 2.72	21.36 ± 3.14	< 0.001
PINP (ng/mL)	49.55 ± 13.44	51.86 ± 15.18	52.80 ± 14.28	0.391

### Comparison of serum oxidative stress factors (SOD, MDA, and NRF2) among the three patient groups

**Figure 2:**
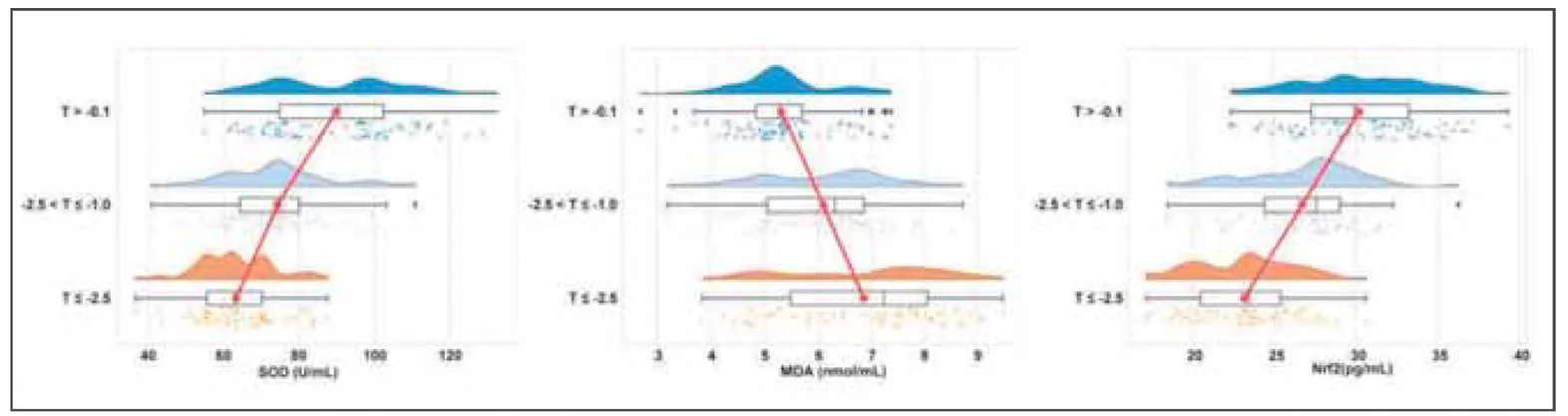
Serum oxidative stress factors (SOD, MDA, and NRF2) among the three patient groups.

Comparative analysis of serum oxidative stress indicators (SOD, MDA, and NRF2) among the three groups was conducted. The results demonstrated that SOD levels were highest in the normal bone mass group (89.88 ± 17.96), decreased in the osteopenia group (74.27 ± 14.51), and were lowest in the osteoporosis group (63.29 ± 10.55) (P < 0.001). This gradual decline in SOD activity from normal bone mass to osteoporosis may indicate reduced antioxidant capacity, leading to increased oxidative stress and subsequently affecting bone metabolism. In contrast, MDA levels showed an opposite trend: the normal bone mass group had the lowest level (5.33 ± 0.91), which increased in the osteopenia group (6.10 ± 1.22) and peaked in the osteoporosis group (6.87 ± 1.50) (P < 0.001). As a marker of oxidative stress, elevated MDA levels may reflect increased oxidative damage and be associated with the progression of osteoporosis. Similarly, NRF2 levels gradually declined, with the highest level observed in the normal bone mass group (33.47 ± 4.09), followed by the osteopenia group (28.38 ± 3.62), and the lowest in the osteoporosis group (26.54 ± 3.19) (P < 0.001). As a key regulator of cellular antioxidant defenses, the NRF2 plays a pivotal role in maintaining redox homeostasis. Diminished NRF2 activity is associated with impaired synthesis of enzymatic antioxidants, including SOD, which may consequently exacerbate oxidative damage (Table 3 and Figure 2).

**Table 3:** Serum oxidative stress factors (SOD, MDA, and NRF2) among the three patient groups.

Indicators	Normal bone mass group<br>(n = 78)	Osteopenia group<br>(n = 62)	Osteoporosis group<br>(n = 56)	*P*-value
SOD (U/mL)	89.88 ± 17.96	74.27 ± 14.51	63.29 ± 10.55	< 0.001
MDA (nmol/mL)	5.33 ± 0.91	6.10 ± 1.22	6.87 ± 1.50	< 0.001
NRF2 (pg/mL)	30.06 ± 3.85	26.70 ± 3.56	23.47 ± 2.94	< 0.001

### Correlation between NRF2-mediated oxidative stress makers and bone mineral density in patients

**Figure 3:**
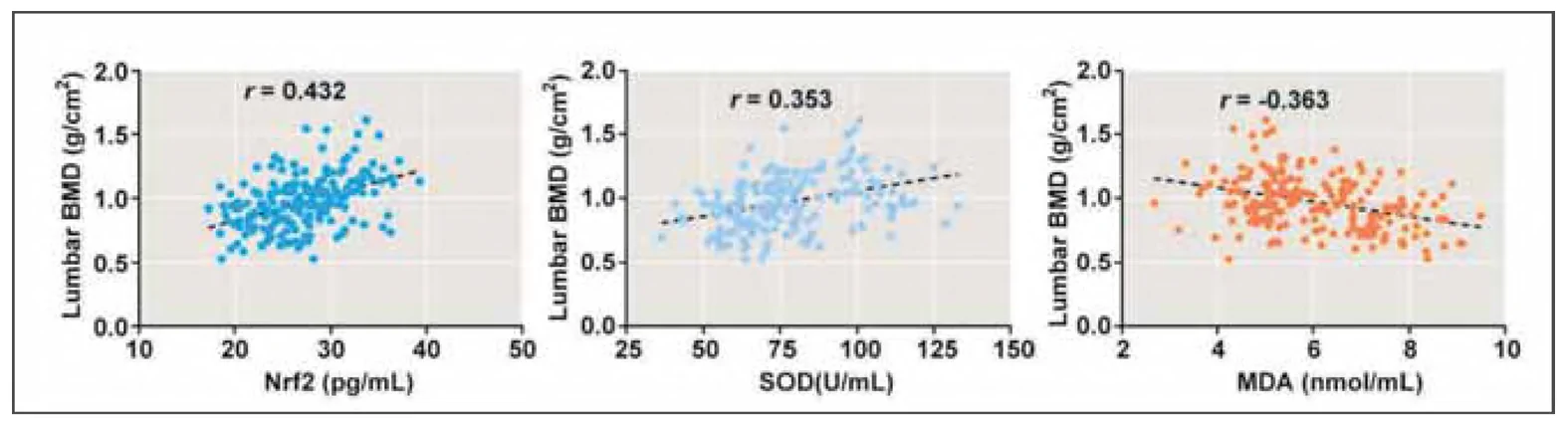
Correlation between serum NRF2, SOD, MDA and BMD.

To investigate the potential associations between serum levels of NRF2, SOD, MDA, and T2DM-OP a Pearson correlation analysis was conducted. The findings revealed a positive correlation between serum levels of NRF2 and SOD with bone mineral density (BMD), while serum MDA levels showed a negative correlation with BMD among all patients (Table 4 and Figure 3).

**Table 4:** Correlation between serum NRF2, SOD, MDA and BMD.

Indicators	BMD	
	*r*	*P*
NRF2	0.432	< 0.001
SOD	0.353	< 0.001
MDA	-0.363	< 0.001

### Multivariate logistic regression analysis of the effects of serum NRF2, SOD, and MDA levels on T2DM-OP

Serum levels of NRF2, SOD, and MDA were included as independent variables, along with gender, age, BMI, and duration of diabetes mellitus as covariates. T2DM-OP was set as the dependent variable (normal or reduced bone mass = 0; osteoporosis = 1) to construct a multivariate logistic regression model. The results showed that decreased serum levels of NRF2 and SOD, as well as increased MDA levels, were risk factors for the development of T2DM-OP (P < 0.05) (Table 5).

**Table 5:** Multivariate logistic regression analysis of the effects of serum NRF2, SOD, and MDA levels on T2DM-OP.

Fator	β	SE	Wald χ^2^	OR (95CI%)	*P*-value
Intercept	10.088	4.570	4.872	-	0.027
NRF2	-0.0.302	0.068	19.802	0.739<br>(0.647-0.845)	< 0.001
SOD	-0.080	0.020	17.149	0.921 (0.886-0.958)<br>	< 0.001
M DA	0.402	0.176	5.210	1.495<br>(1.058-2.111)	0.022

### Diagnostic value of NRF2 in T2DM-OP in the elderly

**Figure 4:**
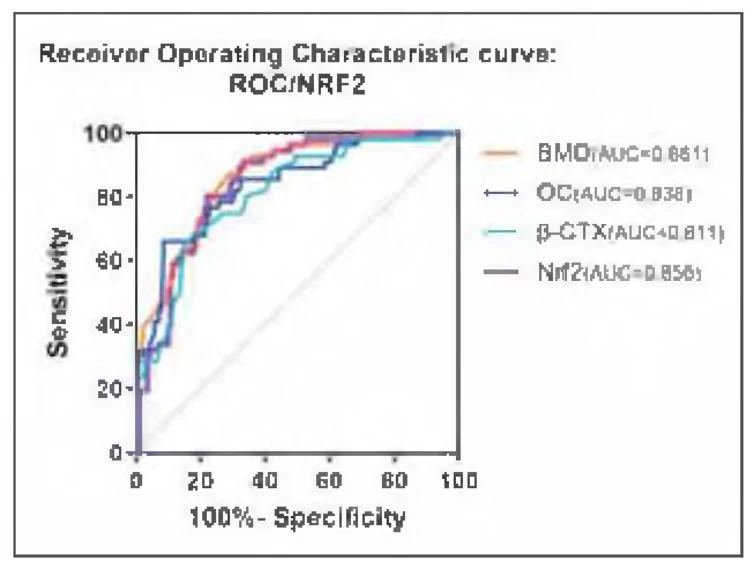
Diagnostic value curve of NRF2 in T2DM-OP in the elderly.

To evaluate the predictive utility of serum NRF2 levels in T2DM-OP ROC curve analysis was employed. The optimal diagnostic threshold for serum NRF2 was identified as 25.73 pg/mL, demonstrating elevated sensitivity (80.36%) and specificity (77.86%) in T2DM-OP prediction, yielding an area under the curve (AUC) of 0.856 (95% confidence interval [CI]: 0.803-0.909; P < 0.001) (Table 6 and Figure 4). Comparative ROC analyses for BMD, OC, and β-CTX revealed the following: BMD: a Uc = 0.861 (95% CI: 0.810-0.912; P < 0.001), sensitivity = 87.50%, specificity = 70.71%, threshold = 0.945 g/cm^2^; OC: AUC = 0.838 (95% CI: 0.775-0.901; P < 0.001), sensitivity = 66.07%, specificity = 91.43%, threshold = 20.95 μg/L; β-CTX: AUC = 0.811 (95% CI: 0.746-0.876; P < 0.001), sensitivity = 66.07%, specificity = 84.29%, threshold = 0.515 μg/L. Collectively, these findings highlight serum NRF2 as a robust biomarker for T2DM-OP diagnosis, comparable to established clinical parameters.

**Table 6:** Diagnostic value of NRF2, BMD, OC, and b-CTX in predicting T2DM-OP.

Indicators	AUC	95CI (%)	Cut-off value	Sensitivity (%)	Specificity (%)
NRF2	0.856	0.803-0.909	25.73 pg/mL	80.36%	77.86%
BMD	0.861	0.810-0.912	0.945 g/cm^2^	87.50%	70.71%
OC	0.838	0.775-0.901	20.95 μg/L	66.07%	91.43%
β-CTX	0.811	0.746-0.876	0.515 μg/L	66.07%	84.29%

## Discussion

A long-term metabolic disorder, diabetes mellitus is identified by the disruption of glucose metabolism [17], which adversely affects the homeostasis of bone minerals such as calcium, phosphorus, and magnesium, resulting in diminished bone mass and structural degradation [18]. Accumulating clinical studies have demonstrated that patients with diabetes exhibit a fracture risk 2- to 3-fold greater than non-diabetic individuals. Notably, over one-third of this population (approximately 35%) concurrently develops diabetes-associated osteoporosis, a metabolic bone disorder characterized by compromised skeletal integrity [19][20]. The risk of decreased hip bone mineral density in patients with T2DM is nearly 1.7 times higher than that in patients without T2DM, and bone density-related fractures are among the most disabling conditions in T2DM [21]. This study focused on elderly patients with T2DM-OP exploring the relationship between NRF2-mediated oxidative stress indicators and the disease. The results revealed several rewarding findings with important implications.

Upon comparing baseline characteristics, the 196 enrolled subjects exhibited no significant differences across the three groups with respect to gender, age, BMI, duration of diabetes mellitus, or various blood biochemical parameters. This established a solid comparable foundation for subsequent investigations of BMD, bone turnover markers, and oxidative stress indicators, effectively eliminating potential confounding effects from these variables and enhancing the reliability of our findings. The comparison of BMD and bone turnover markers among the three groups revealed that lumbar spine BMD significantly decreased with progressive bone loss. In the osteoporosis group, there was a significant elevation in the bone resorption marker β-CTX and the bone formation marker OC, accompanied by a marked reduction in vitamin D levels. No significant difference was detected in the levels of procollagen type I N-terminal propeptide (PINP). These findings elucidate the bone metabolic characteristics associated with changes in bone mass among patients with T2DM, underscoring the presence of high-turnover osteoporosis. The observed reduction in vitamin D levels further suggests its potential role in bone loss. Insulin deficiency impacts calcium transport and vitamin D metabolism, leading to decreased serum calcium levels and heightened osteoclast activity. Notably, in patients with diabetes mellitus who also experience compromised liver and kidney function, the active form of vitamin D diminishes, resulting in reduced intestinal calcium absorption [22]. These findings align with prior research and substantiate the observed pattern of bone metabolism abnormalities in patients with T2DM. They offer a metabolic basis for further exploration into the role of oxidative stress within this demographic.

Oxidative stress, a key pathogenic factor mediating pancreatic β-cell damage under hyperglycemic conditions, is recognized as a common pathological mechanism underlying diabetes mellitus-associated complications, including OP, periodontal disease, coronary heart disease, and atherosclerosis [23]. As a crucial regulator of the oxidant-antioxidant balance, NRF2 is essential for maintaining redox homeostasis by preventing the binding of Keap1 and regulating the expression of antioxidant genes [24]. In biological systems, free radicals act on membrane lipids to initiate lipid peroxidation, producing MDA as a final oxidative product. MDA demonstrates cytotoxic characteristics and possesses the ability to induce crosslinking between proteins and nucleic acids. SOD, a vital antioxidant metalloenzyme, catalyzes the dismutation of superoxide anions into molecular oxygen and hydrogen peroxide, thereby playing a critical role in maintaining oxidative-antioxidant homeostasis. In clinical contexts, MDA and SOD are widely employed as biomarkers for assessing oxidative stress-related damage [25][26]. Regarding serum oxidative stress factors, SOD activity gradually decreased as bone mass declined, while MDA levels progressively increased. Reduced SOD activity, as a primary antioxidant enzyme, indicates impaired antioxidant capacity and enhanced oxidative stress in the body. Elevated MDA, a marker of oxidative stress, reflects aggravated oxidative damage. NRF2, a key transcription factor in the antioxidant response, exhibits declining levels that affect the expression of antioxidant enzymes, exacerbating oxidative stress. The osteoporosis group showed lower NRF2 expression compared to the osteopenia and normal bone mass groups, suggesting that low NRF2 expression may be correlated with the development and progression of T2DM-OP Low NRF2 expression has been linked to oxidative stress- related diseases, as NRF2 primarily regulates intracellular antioxidant responses and the expression of detoxifying enzymes. Reduced expression indicates uncontrolled oxidative stress. A study has found that ovariectomy-induced osteoporotic rats exhibit significantly decreased levels of SOD, along with increased serum OC, alkaline phosphatase, MDA levels, and RANKL expression [26]. These findings reveal the dynamic changes in oxidative stress during the progression from reduced bone mass to osteoporosis in patients with T2DM. Oxidative stress plays a critical role in high glucose-induced osteoblast apoptosis [27]. Oxidative stress is one of the major causes of osteoblast apoptosis [28]. Therefore, detecting and evaluating the expression of these oxidative stress markers is of great significance for the diagnosis and treatment of T2DM complicated by osteoporosis, as it helps reduce the occurrence of oxidative stress.

From a clinical application standpoint, NRF2 has the potential to function as a biomarker for evaluating the risk in patients with T2DM-OP Pearson correlation analysis and multivariate logistic regression analysis have elucidated the significant association between NRF2-mediated antioxidant stress mechanisms and T2DM-OP In the patient cohort, serum NRF2 and SOD levels demonstrated a positive correlation with BMD, whereas serum MDA exhibited a negative correlation with BMD. The multivariate logistic regression model identified that reduced levels of serum NRF2 and SOD, coupled with increased MDA levels, may constitute risk factors for T2DM-OP with statistical significance (P < 0.05). In addition, the ROC curve analysis demonstrated that serum NRF2's ability to predict T2DM-OP was equivalent to that of bone turnover markers used clinically. In clinical practice, regular monitoring of these indicators in patients with T2DM could help early identification of high-risk individuals, allowing for targeted interventions such as lifestyle modifications and antioxidant supplementation to delay or even prevent the onset and progression of OP Furthermore, targeting NRF2 for drug development-activating the NRF2-mediated antioxidant pathway, enhancing SOD activity, and reducing MDA levels-may provide a novel and effective therapeutic strategy for T2DM-OP

Nonetheless, this study possesses certain limitations. The sample was derived from a specific region, potentially restricting the geographical applicability of the findings and necessitating further validation to assess their generalizability. Given its design as a small-sample cross-sectional study, it is incapable of establishing causal relationships between the variables. Future research should incorporate multicenter, large-scale prospective studies to further examine these associations. Additionally, while the study identified a connection between the NRF2-mediated antioxidant stress mechanism and T2DM-OP the precise molecular mechanisms remain inadequately elucidated and warrant further investigation at both the cellular and molecular levels.

In summary, this investigation elucidates the critical therapeutic implications of NRF2-dependent antioxidant pathways in mitigating oxidative damage among geriatric populations with T2DM-OP While further validation is required to address current limitations, these results lay the groundwork for novel approaches in translational research and clinical translation, thereby advancing the development of personalized interventions and disease-modifying therapies for T2DM-OP management.

## Dodatak

### Acknowledgments

Not applicable.

### Funding

State Key Laboratory of Pathogenesis, Prevention and Treatment of High Incidence Disease in Central Asia &The First People's Hospital of Kashi Fund (Number: SKL-HIDCA-2024-KY7).

### Competing interests

The authors have no conflicts of interest to declare.

### Data available

Data is available from the corresponding author on request.

### Consent to participate

Written informed consent was obtained from each subject.

### Consent to publish

Written informed consent for publication was obtained from all participants.

### Ethics statement

The present study was approved by the Ethics Committee of The First People's Hospital of Kashi (No.202209XJ85) and written informed consent was provided by all patients prior to the study start. All procedures were performed in accordance with the ethical standards of the Institutional Review Board and The Declaration of Helsinki, and its later amendments or comparable ethical standards.

### Author's contribution

Conceptualization, Reyila Fulati; data curation, Guzainu Ailiyasi and Renagu Baihetiya; investigation, Reyila Fulati and Gulizhaer Tulak; formal analysis, ZhenHua Li and Gulizhaer Tulake; methodology, JiWei Yu and XiuFang Wang; writing—original draft preparation, Reyila Fulati; writing—review and editing, ZhenHua Li and Gulizhaer Tulake. All authors have read and agreed to the published version of the manuscript.

### Conflict of interest statement

All the authors declare that they have no conflict of interest in this work.
